# Supplementary material for: To assess the determinants of family planning uptake among women of reproductive age in rural settings, Morogoro Region, Tanzania. Protocol for a cross-sectional study
Source: PLoS One. 2022 Apr 15;17(4):e0267020. doi: 10.1371/journal.pone.0267020 (PMC9012382; doi:10.1371/journal.pone.0267020)
Supplement: S1 Appendix — (DOCX) [file pone.0267020.s001.docx]

S1 Appendix: Questionnaire

Preamble

Dear

participants, we would like to thank you for agreeing to take part in this important piece of research. We are trying to assess determinants of family planning uptake among women of reproductive age in rural areas, Morogoro region*.* The findings of this study will work out as the starting point for the regional health management team and stakeholders of maternal health services, to put in place strategies to improve uptake of family planning in the region so as to achieve the national goal of 60% family planning services utilization, reduction of maternal deaths by a three-quarter. Therefore, we honestly request your collaboration in attending to these questions responsibly because your truthful response is of great importance for the success of this study.

**Introduction**

Dear participant, you are humbly requested to participate in this study which aims to *“assess determinants of family planning uptake among women of reproductive age in rural areas, Morogoro region.”* Your participation is highly valued and appreciated. I assure you that all your comments will remain confidential. The interview will take you few minutes of less than 15 minutes.

Questionnaire Number……………….

Date of Interview…………………….

Name of Interviewer…………………

District……………………………….

Ward………………………………….

Street/Village…………………………

**PART I: DEMOGRAPHIC INFORMATION**

1. How old are you? ………………………………

2. What is your tribe?

- 1. Luguru
  2. Kaguru
  3. Pogoro
  4. Others

3. Please **tick** against your highest level of education?

1. Non formal
2. Primary
3. Secondary
4. Certificate
5. Diploma
6. University

4. What is your current (Major) occupation status (multiple response – employees)?

1. Employed public sector
2. Employed private sector
3. Employed permanent contract
4. Employed temporary contract

1. Business
2. Peasant/farming
3. Livestock keeper
4. Others (name)

5. What is your marital status?

1. Single (Not in relationship)
2. Married
3. Cohabiting
4. Separated/ Divorced
5. Widowed
6. Others.

6. If married, What type of marriage are you living in?

1. Monogamy b. Polygamy

7. At which age did you get married? Specify..............

**PART II: FAMILY PLANNING UPTAKE OF THE PARTICIPANTS**

8. Have you ever given birth?

a) Yes b) No

9. At which age did you get your first child? Specify …………

10. How many times have you given birth? Specify ……………

11. How many children are alive? Specify …………

12. Which one describe the sex for your children?

1. All male
2. All female
3. More males than female
4. More female than male
5. Equal number of males and female.

13. When did you deliver your previous child? Specify……….

14. How did you deliver your previous child?

a. By Caesarian section b. By Spontaneous Vertex Delivery.

15. How old is your previous child? Specify………………

16. Where did you give birth for the previous child?

1. Health facility
2. Home
3. Birth Before Arrival.

17. Did you attend ANC services in your previous pregnancy?

a. Yes b. No

18. Were you counseled about FP methods use during ANC visits?

1. Yes b. No

19. Were you counseled about FP methods use before discharge following childbirth?

1. Yes b. No

20. Would you like to space your children?

1. Yes
2. No

21. If yes, at what interval would you like to space your children? Specify (in years) .

22. How many children would you like to have? Specify………

23. Who decides the number of children to have in the family?

1. I myself
2. I and my husband/partner
3. My husband/partner alone
4. My in laws
5. Others. Specify……

24. Do you know about FP method?

a. Yes b. No

25. Where did you get information regarding family planning methods?

1. Health facility
2. Radio/TV
3. IEC materials (pamphlets)
4. Relatives
5. Neighbors

26. Which methods of FP do you know?

1. Pills
2. Injectable
3. IUCD
4. Implant
5. Male condom
6. Female condom
7. Sterilization
8. LAM
9. Periodical Abstinence
10. Withdrawal
11. Cycle beads
12. . None
13. Others

27. Have you ever used any family planning technique for the past 3 months?

1. Yes
2. No

28. If yes, which method have you ever used among the following?

1. Pills
2. Injectable
3. IUCD
4. Implant
5. Male condom
6. Female condom
7. Sterilization
8. LAM
9. Periodical Abstinence
10. Withdrawal
11. Cycle beads
12. None
13. Others. Specify…….

29. If you are currently using any FP method, what made you to prefer the method you are using?

1. Safety
2. Effectiveness
3. It is accepted by my religion
4. Few sides effect
5. The only method I know
6. Only method available
7. Affordable
8. Accessible
9. Don’t know.

30. If you have ever used or using any FP method, where did you obtain it?

1. Health facility (clinic)
2. Private organization
3. From local shops
4. Other: Specify ………

31. If no, give the reasons of not using family planning (specify)….

32. If no, to question above, do you plan to use family planning in the future?

1. Yes
2. No

33. If yes to question above which method among the following do you plan to use?

1. Pills
2. Injectable
3. IUCD
4. Implant
5. Male condom
6. Female condom
7. Sterilization
8. LAM
9. Periodical Abstinence
10. Withdrawal
11. Cycle beads

34. Why would you prefer to use FP method; you have mentioned above?

1. Safety
2. Effectiveness
3. It is accepted by my religion
4. Few sides effect
5. The only method I know
6. Only method available
7. Affordable
8. Accessible
9. Don’t know

35. If you are using FP method do you receive any support from your husband/partner?

1. Yes
2. No

36. If yes, what kind of support on FP do you get from your husband/partner? (specify)…………

37. If your husband/partner does not support you, what do you think might be the reasons? Specify…………….

38. Does your husband/partner know that you are using FP method?

1. Yes
2. No

39. Do other family members support you on the use of family planning

1. Yes
2. No

40. If Yes; What kind of support on FP do you get from other family members? Specify……………

41. If other family member does not support you on FP use, what do you think might be the reasons? Specify………….

42. Does the community you are living in support family planning services?

1. Yes b. No

43. If Yes; What kind of support on FP do you get from community (specify)…………

44. If your community does not support you, what do you think might be the reasons? Specify………….

45. Who decided on the current family planning method you are using?

1. I Myself
2. My husband/partner
3. I and my husband/partner
4. Healthcare provider
5. My in – laws
6. Others. Specify….

**PART III: CULTURAL FACTORS AFFECTING PARTICIPANTS’ UPTAKE OF FAMILY PLANNING METHODS**

46. What beliefs do you have regarding family planning methods? ***(Encircle All That Apply)***

1. They reduce libido
2. They make private parts wet most of the time
3. They can cause cancer
4. They increase promiscuity
5. Against religion believes

47. Are there any traditional and cultural beliefs that prohibit FP methods use in your community?

1. Yes
2. No

48. If yes what are they? Mention two

i. ………………………………………………………….

ii…………………………………………………..………

49. Is there any health care facility in your place of residence?

1. Yes

b. No

50. Can you estimate the time do you take to reach the health facility for family planning services? Specify (in hours) ………………….

51. Does the facility provide FP services?

1. Yes
2. No

52. What kind of FP services do you get from the facility? Specify………

**PART IV: CHALLENGES OF CLIENTS ON FAMILY PLANNING UPTAKE**

53. Are you aware of the side effects of FP methods?

a) Yes

b) No

54. What among the following are the side effects associated with FP methods use? *(Encircle All That Apply)*

1. Spotting
2. Irregular bleeding
3. Heavy bleeding
4. No monthly bleeding
5. Weight gain
6. Weight loss
7. Excess vaginal secretions
8. Loss of libido
9. Frontal headache
10. Backache
11. Lower abdominal pain
12. Mood change

55. Are there any existing myths or misconceptions in your society regarding FP methods?

- 1. Yes b. No

56. If your answer is yes, what are they? Mention any two:

1. ………………………………………………………………………….
2. ………………………………………………………………………….

57. What measures can be taken to improve FP uptake? Mention any two:

1. ……………………………………………………………………………
2. ……………………………………………………………………………..

58. What challenges did you face while seeking for FP services at the health facility? Specify………………….

******THANKS FOR YOIUR PARTICIPATION*******
