# Supplementary material for: To assess the determinants of family planning uptake among women of reproductive age in rural settings, Morogoro Region, Tanzania. Protocol for a cross-sectional study
Source: PLoS One. 2022 Apr 15;17(4):e0267020. doi: 10.1371/journal.pone.0267020 (PMC9012382; doi:10.1371/journal.pone.0267020)
Supplement: S2 Appendix — (DOCX) [file pone.0267020.s002.docx]

S2 Appendix: In-Depth Interview Guide

Name of interviewer……………………………………….

Position of the interviewer…………………………………

Date of interview……………………………………………

Mobile number of the interviewee ………………………….

Residence of the interviewee: Ward……………. District…………………………….

Good morning. I am ________ *(self-introduction).*

This interview is being conducted to get your input about the challenges/barriers you face in using family planning methods.

If it is okay with you, I will be tape recording our conversation. The purpose of this is so that I can get all the details but at the same time be able to carry on an attentive conversation with you. I assure you that all your comments will remain confidential. I will be compiling a report which will contain all staff comments without any reference to individuals. If you agree to this interview and the tape recording, please sign this consent form. *(Produce the consent form to her to sign).*

1. Concerning FP methods: I would like to start by hearing from you briefly about what you know about family planning methods?
2. Have you ever used FP methods? If yes which type of FP method currently you are using?
3. Which type of FP method used in your place?
4. What are the benefits of using FP methods?
5. Concerning side effects of FP methods: Do you know side effects of FP?
6. How do side effects prevent you from effectively using FP methods?
7. Concerning male partner involvement: Are you currently living with partner?
8. In what ways does your male partner support you in FP uptake?
9. Concerning traditions and cultural beliefs: How do existing traditional and cultural beliefs in your community (if any) affects/is a challenge to you in utilizing FP methods?
10. Concerning existing myths (misconceptions): What is the existing myth about FP methods?
11. How (if any) myths about FP methods uptake prevent you from utilizing the service?
12. Concerning religious beliefs: What is the role of your religious beliefs in FP methods uptake?
13. How your religious beliefs do is a challenge to you in utilizing FP methods?
14. Concerning decision making ability of the woman: what do you say about decision making
15. What measures do you think can be taken to improve FP uptake?
16. Is there any other information about the challenges blocking you from using FP methods that you think would be useful for me to know?

***Thank You Very Much for Your Active Participation in This Interview.***
