# Supplementary material for: To assess the determinants of family planning uptake among women of reproductive age in rural settings, Morogoro Region, Tanzania. Protocol for a cross-sectional study
Source: PLoS One. 2022 Apr 15;17(4):e0267020. doi: 10.1371/journal.pone.0267020 (PMC9012382; doi:10.1371/journal.pone.0267020)
Supplement: Attachment — Submitted filename: PLOS ONE Review.docx [file pone.0267020.s003.docx]

**Review**

**PLOS ONE**

**Review of PLOS ONE: PONE-D-21-12824**

Article Type: Study Protocol

**Title:** Assessing Determinants of Family Planning Use among Women of Reproductive Age in Rural Areas, Morogoro, Tanzania. Mixed Cross-Sectional Protocol Study.

**Authors:** margareth danielistan machange.

**Comments to the authors**

I enjoyed reading the study protocol, and it is an important topic and well presented. This protocol manuscript is also well written. However, I do have some points that require the authors' attention.

- The discussion section must be added. This section should include any issues involved in performing the study that are not covered in other sections. These include: limitations of the study design & dissemination plans.

Sincerely,

**Reviewer:** Tesfalem Tilahun Yemane
