# Supplementary material for: To assess the determinants of family planning uptake among women of reproductive age in rural settings, Morogoro Region, Tanzania. Protocol for a cross-sectional study
Source: PLoS One. 2022 Apr 15;17(4):e0267020. doi: 10.1371/journal.pone.0267020 (PMC9012382; doi:10.1371/journal.pone.0267020)
Supplement: Attachment — Submitted filename: PROTOCOL REVIEWERS RESPONSE Plos.doc [file pone.0267020.s004.doc]

**POINT-BY-POINT RESPONSES TO REVIEWERS’ COMMENTS**

We would like to take this opportunity to thank you all, editors and reviewers from PLOS ONE We have appreciated all the comments given in order to improve the manuscripts. We have thoroughly responded to all reviewers’ comments. The table below is a summary of responses for the reviewers.

|  | **Comments to the authors** | **Authors’ Response** |
| --- | --- | --- |
|  |  | Authors had revised the whole document for grammar check and clarity |
| 1 | Please include additional information regarding the survey or questionnaire used in the study and ensure that you have provided sufficient details that others could replicate the analyses. For instance, if you developed a questionnaire as part of this study and it is not under a copyright more restrictive than CC-BY, please include a copy, in both the original language and English, as Supporting Information. If the original language is written in non-Latin characters, for example Amharic, Chinese, or Korean, please use a file format that ensures these characters are visible. | The questionnaire description has been revised. The questionnaire has been adapted from the Demographic Health Surveys questionnaire. We only selected the questions related to family planning for this particular study. Minor modification has been done.  The questionnaire has been uploaded as additional file (Appendix 1) (Page 6) |
| 2 | Please state whether you validated the questionnaire. Please provide details regarding the validation group within the methods section. | The questionnaire was not validated because it was extracted from a standard questionnaire that is normally used for country wide survey.  However minor modifications has been made |
| 3 | Please include a copy of the interview guide to be used in the study, in both the original language and English, as Supporting Information, or include a citation if it has been published previously. | A copy of interview guide has been added and uploaded as additional file |
